# Polymorphism of Selected Regions of *Ovar-*MHC and the Health Status of the Ovine Mammary Gland

**DOI:** 10.3390/ani10122325

**Published:** 2020-12-07

**Authors:** Wiesław Piotr Świderek, Joanna Gruszczyńska, Anna Winnicka

**Affiliations:** 1 Department of Animal Genetics and Conservation, Institute of Animal Sciences, Warsaw University of Life Sciences—SGGW, Ciszewskiego 8, 02-786 Warsaw, Poland; wieslaw_swiderek@sggw.edu.pl; 2 Department of Pathology and Veterinary Diagnostics, Institute of Veterinary Medicine, Warsaw University of Life Sciences—SGGW, Nowoursynowska 159C, 02-776 Warsaw, Poland; anna_winnicka@sggw.edu.pl

**Keywords:** sheep, microsatellite polymorphism, *Ovar-*MHC, mastitis, lymphocytes

## Abstract

**Simple Summary:**

A common cause of economic losses in sheep breeding is udder diseases (*mastitis*). The main cause of disease is pathogenic microbes. Their infection adversely affects the quality and quantity of milk and the effects of lamb rearing. Methods of treating this disease so far haven’t brought satisfactory results. Therefore, in this study, an attempt is made to search for natural resistance to udder diseases. The research is carried out on sheep of the Polish Heath and Polish Lowland breeds. Genetic variability of the major histocompatibility complex (MHC) genes are analyzed. MHC molecules enable the recognition of the pathogen and activate other cells of the immune system (T and B lymphocytes) to organize the defense. The content of somatic cells (SCC) in milk (as an indicator of udder health) and the percentage of lymphocyte subpopulation are also assessed in the examined sheep. Among the many alleles of the MHC genes (*OLADRB1, OLADRB2, OMHC1*), two of them, 488 bp (*DRB1*) and 284 bp (*DRB2*) in length, were more frequently reported in sheep, which milk contained <200 × 10^3^/mL SCC, while in carriers of the 508 bp (*DRB1*) and 272 bp alleles (*DRB2*) SCC level in milk was significantly higher (>200 × 10^3^/mL). The obtained results justify the need for further research aimed at searching for genetic markers related to resistance to mastitis in sheep.

**Abstract:**

Udder diseases (*mastitis*) are a serious cause of economic losses in sheep breeding as they have a negative impact on lamb rearing and the quality of dairy products. Thus far, progress in treatment and prevention of these diseases has been insufficient—giving ground for searching possibilities of using natural immunity to combat mastitis. This study aims to assess the relationship between the microsatellite polymorphism of selected *Ovar-*MHC genes and the health status of the mammary gland of sheep. The research is carried out on sheep of the Polish Heath and Polish Lowland breeds. In ovine milk, the SCC and the percentage of the lymphocyte subpopulation are assessed. Based on genomic DNA, molecular analysis of the *Ovar-*MHC gene fragments (*OLADRB1, OLADRB2, OMHC1*) polymorphism is performed. Significant differences are found in SCC level and the percentage of lymphocytes (CD4, CD8, CD19) in the milk depending on the alleles of the *Ovar-*MHC genes. Alleles of 488 bp (*DRB1*) and 284 bp (*DRB2*) are found more frequently in sheep, which milk contained <200 × 10^3^/mL SCC, while in carriers of the 508 bp (*DRB1*) and 272 bp (*DRB2*) alleles, SCC level in milk is significantly higher (>200 × 10^3^/mL). The obtained results justify the need for further research to better understand the genetic basis of mastitis, and to search for effective molecular markers that can be used in breeding practice.

## 1. Introduction

Inflammation of the udder (mastitis) is a major cause of economic losses in sheep farming. During udder infection, the content of certain milk components (casein, lactose, fat) and microelements in milk (calcium, phosphorus, potassium, magnesium) decreases, while the number of somatic cells, including leukocytes (mainly neutrophils), increases. Losses in milk production and changes in its physicochemical properties caused by mastitis adversely affect primarily lamb rearing (growth and development, diseases, falls), as well as the quality of dairy products [1,2,3,4,5]. The main causes of udder diseases are pathogenic microorganisms—mainly staphylococci and streptococci, much less often bacilli (Gram+, Gram−), and mycoplasmas [6,7,8,9,10,11,12,13,14,15,16]. In addition to pathogenic microorganisms, the development and course of mastitis are also influenced by genetic factors (individual, breed, and species-specific), biological factors (number of lactations and their phase, general health condition of the organism), and environmental factors (nutrition, care, type of premises, zoohygienic conditions) [17,18,19]. Immunity is a complex physiological process in which the major histocompatibility system plays a fundamental role. This system includes many polymorphic genes coding for the surface structures of the body’s cells, known as MHC molecules. Class I MHC molecules present antigens to cytotoxic T lymphocytes (CD8^+^), while class II molecules initiate an immune response by presenting antigens to helper T cells (CD4^+^). One of the consequences of this presentation is the stimulation of B lymphocytes (CD19^+^) to proliferate and produce antibodies specific for given antigens. Many studies clearly show that during bacterial infections of the mammary gland in ruminants, due to the increased permeability of the udder epithelium, there is an increased flow of blood serum components and leukocytes from the blood into the milk, of which the most numerous are neutrophils [20]. At the same time, there is a significant decrease in the percentage of T lymphocytes (total CD2^+^ and the CD4^+^ subpopulation, CD8^+^) and B lymphocytes (CD19^+^), as well as cells with the MHCII receptor [21,22,23,24]. Several studies found an increase in the percentage of lymphocyte subpopulations during bacterial infections in cattle [25,26,27]. Many authors used microsatellite sequences (STR) to assess the variability of the MHC genes in ruminants. Molecular studies conducted in sheep have shown significant STR polymorphism within the *Ovar-DRB1* and *OMHC1* genes and the *Ovar*-*DRB2ps* pseudogene [28,29,30,31,32,33,34,35,36,37]. Further studies showed that some of the microsatellite alleles of MHC genes may be associated with sheep resistance to diseases, such as sprays [38], leukemia [39], or parasitic invasions [40,41,42,43,44]. Correlation between the polymorphism of the selected *Ovar-*MHC genes and the yield and quality of sheep milk, as well as weight gain of lambs, was also noted [30,34,45,46].

The aim of this study was to evaluate the relationship between the polymorphism of selected *Ovar-*MHC genes and the health status of the mammary gland of sheep, expressed in the number of SCC and the percentage of lymphocyte subpopulation in milk.

## 2. Materials and Methods

### 2.1. Animals and Samplings

This research was carried out on 150 sheep of the Wrzosówka breed (Polish Heath Sheep—PHS) and on 154 lowland sheep of the Żelaźnieńska breed (Polish Lowland Sheep—PLS). Both breeds are included in the national conservation breeding program. PHS sheep are characterized by a delicate body structure, easy adaptation to even very difficult environmental conditions, good viability, fertility, and resistance to diseases [47,48]. PLS sheep were produced by crossbreeding, enhancing the regional Łowicz sheep. As a result of many years of breeding work, quite massive sheep were obtained, characterized by good meat and wool performance, and high fertility [47,48]. Research material was collected once from mothers of both breeds, in the 4th week of lactation (first or second), always at the same time of the day (morning), 2 h after their lambs were weaned. Milk samples (20 mL) were taken from each half of the udder separately into two test tubes. At the same time, 8 mL of blood (tubes with K2EDTA) were collected from the same mothers from the external jugular vein for molecular testing. Blood samples were obtained from a veterinary clinic that collected sheep blood for veterinary diagnosis. In this case, in accordance with the applicable legal regulations contained in the Journal of Laws of 2015 (item 266, art. 1 section 2 point 2), the consent of the Ethics Committee is not required.

### 2.2. Somatic Cell Count

The SCC in 1 mL of sheep milk was assessed using a Somacount 150 counter (Bantley Instruments Inc., Chaska, MN, USA). Test samples were prepared in accordance with the manufacturer’s instructions. Udder’s health condition was assessed based on the SCC. Based on the literature data [49,50,51,52,53], the level of 200 × 10^3^/mL was adopted as the physiological norm. SCC above this level was considered a probable effect of subclinical inflammation of the mammary gland, SCC below 200 × 10^3^/mL was considered a sign of udder health.

### 2.3. Lymphocyte Subpopulation Level Determination

Chilled milk samples (15 mL) were diluted with PBS (15 mL) and centrifuged for 20 min in a refrigerated centrifuge (2000 rotations/min), after which the fat and supernatant were poured off. The obtained pellet was washed three times with PBS with heparin (5 U heparin/1 µL PBS) for 10 min followed by centrifugation each time. After discarding the supernatant, the isolated cells were resuspended in 400 µL of PBS. Identification of sheep milk immune system cells (ISCs): CD2, CD4, CD8 (T cells), CD19 (B cells), and MHC II (B cells, APC) positive cells was performed by flow cytometry using a monoclonal antibody anti-: CD2 (clone MUC2A), CD4 (clone GC1A), CD8 (clone CACT130A), CD19 (clone BAQ44A), MHC II (clone TH14B) (VMDR Inc. Pullman, USA) and fluorescent dyes (fluorescein—FITC or phycoerythrin—PE) (Medac GmbH, Wedel, Germany). According to the developed procedure [54,55], to each tube, 50 μL of the cell suspension and 1 μL of the appropriate monoclonal antibody were added. After discarding the supernatant, 1 μL fluorescein-labeled (FITC) polyclonal antibodies were added to the test tubes (except for the control sample) and incubated for 15 min at room temperature. The remaining unbound antibodies were washed out twice with 1 mL buffer (PBS, 10% ACD, 0.2% NaN3, 10 mM K_2_EDTA) and centrifuged. After discarding the supernatant, a second antibody was added and incubated for 15 min in the dark. After that, it was washed again twice with PBS buffer (1 mL) and centrifuged. After discarding the supernatant, 1 μL phycoerythrin (PE) labeled antibodies were added and incubated (15 min). Then 1 mL of Lysate (FACS-lysing solution) was added to all tubes. After incubation (10 min) and centrifugation, it was washed twice more with PBS buffer and centrifuged again. After discarding the supernatant, the labeled cells were resuspended in 200 µL of PBS with 0.5% formaldehyde. The results were read out and analyzed in detail using a FACScalibur flow cytometer (Becton-Dickinson GmbH, Heidelberg, Germany) and the SimulSET and CellQuest programs.

### 2.4. Genomic DNA Extraction and Molecular Analysis

Genomic DNA was isolated from sheep blood with the phenol-chloroform method. The concentration and purity of the isolated DNA were determined with a Gene Quanty spectrophotometer (Pharmacia). The tested fragments of the *Ovar-DRB1* gene (exon 2, intron 2, MHC class II), *OMHC1* (MHC class I), *Ovar-DRB2ps* (intron 5, MHC class II) were amplified using the classic PCR technique [45,56,57]. Identification of microsatellite alleles was performed using an ABI PRISM^TM^ 310 Genetic Analyzer (Perkin Elmer, Foster City, CA, USA) and analysed by Gene Scan Analysis software version 2.1 (Perkin Elmer, Foster City, CA, USA). Based on the results of the molecular analysis for each breed of sheep, the parameters describing genetic variation were estimated using the Cervus 3.0.7 program [58]: Frequencies of alleles and genotypes (ALFreq program 1.03. [59]) of microsatellite sequences (fragments of *Ovar-DRB1* genes, *OMHC1*, *Ovar-DRB2ps*), coefficients: Observed heterozygosity (H_O_) and expected heterozygosity (H_E_), Polymorphic Information Content (PIC), compliance of genotype distribution with the Hardy-Weinberg law (chi^2^ test). The Fis and Fis parameters for multilocus (10,000-permutation) were estimated according to the formulas of Weir and Cockerham [60] and Robertson and Hill [61]. Moreover, with the help of the GENETIX 4.05.2 program [62], the genetic distance [63] between the studied breeds was estimated, and (on the basis of the Factorial Correspondence Analysis—CA [64]) graphs were made showing the relationships between genotypes individual multilocus. The Bayesian clustering method implemented in the program STRUCTURE 2.3.4 [65] was used with an admixture model and correlated allele frequencies to detect substructure in the data, assign individuals to cluster, and identify potentially admixed genotypes. The optimal number of clusters (K) was set by running the program from K = 1 to K = 5, with 10 repetitions of 1,000,000 MCMC chain steps after a burn-in period of 1,000,000 steps for each K. STRUCTURE results were visualized in STRUCTURAL HARVESTER [66] implementing the method of Evanno et al. [67]. Graphical output was performed in DISTRUCT 1.1. [68].

### 2.5. Statistics

Statistical analysis employed non-parametric methods as variables did not comply with the requirements of normal distribution. The significance of differences between the compared groups was assessed using non-parametric Mann-Whitney or Kruskal-Wallis tests (depending on the number of levels). All calculations were made using the Statistica 13.3 program.

## 3. Results

Detailed results of the molecular analysis of selected *Ovar*-MHC regions: *Ovar-DRB1, OMHC1, Ovar-DRB2ps* are summarized in Table 1. Different frequency of individual alleles of *Ovar-*MHC genes both within and between races was noted. The 488 bp allele (*Ovar-DRB1*), which was most common in sheep, was distinguished by over seven times lower frequency in PLS. In turn, the 566 bp and 508 bp alleles in PLS were noted 2–3 times more often than in PHS. In both breeds of sheep, six out of the seven identified *OMHC1* alleles were of identical length (from 188 bp to 202 bp), the allele of 208 bp was found only in PHS sheep. In the case of the 192 bp and 196 bp allele frequencies, large interracial differences were observed. High frequency of a given allele in one breed of sheep corresponded to low frequency in the other breed (allele 192 bp in PHS-32.3% in PLS-6.8%, allele 196 bp in PHS-4.0% in PLS-25.6%). The difference in frequency between the remaining alleles in PHS and PLS was much smaller. Out of the seven identified alleles of the *Ovar-DRB2* pseudogene, two alleles: 272 bp and 276 bp, were found only in the PLS breed, while the 284 bp allele only in the PHS breed.

**Figure 1:**
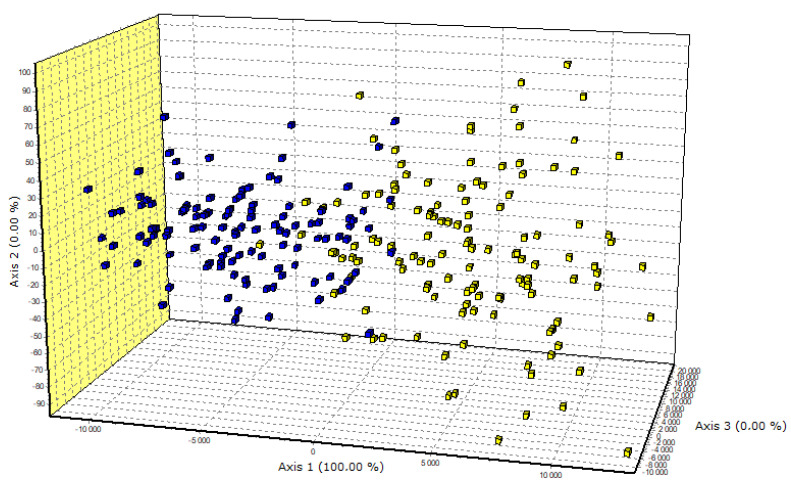
The factorial correspondence analysis was performed in GENETIX on the base of 3 *Ovar-*MHC *loci*. Each of the three axis: Axis 1, Axis 2, and Axis 3, describes the maximum genetic variation between individuals, where the first axis (Axis 1) always describes its largest part. Genotype distribution between the two breeds: PLS—Polish Lowland Sheep (blue); PHS—Polish Heath Sheep (yellow).

**Figure 2:**
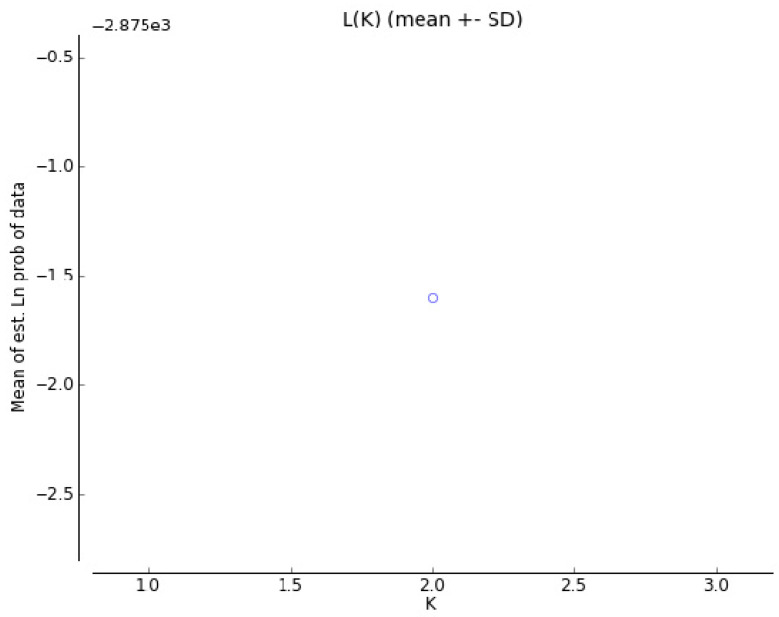
The posterior probability Ln (K) of the data.

**Figure 3:**
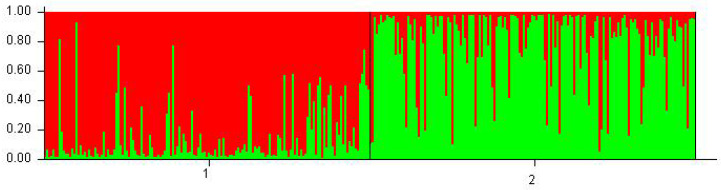
STRUCTURE analyses performed of Bayesian clustering analysis of the two breeds PHS (**1**) and PLS (**2**) analyzed on the basis of three *Ovar-*MHC *loci*. Each individual is represented by one vertical bar that is divided into segments representing the proportion of memberships to the respective breeds. The results are displayed for two (K = 2) suggested clusters.

The results of the microsatellite polymorphism analysis of the three *Ovar-DRB1*, *OMHC1*, and *Ovar-DRB2ps loci* were also used to estimate the parameters of genetic variation (Table 2). Compared to PHS, PLS showed lower Ho coefficients for the *Ovar-DRB1* and *Ovar-DRB2ps loci*, and a higher value for *OMHC1*. However, the values of H*_E_* coefficients for all analyzed *loci* in both breeds of sheep were at a similar level (0.74–0.78). The PIC values were slightly lower than the H values. The presented parameters indicate that the PHS breed was characterized by greater heterozygosity than the PLS breed; however, the differences in the values of these parameters between the tested breeds (except for *Ovar-DRB1*) were small. This is also confirmed by statistics. For the *Ovar-DRB1* and *OMHC1 loci* in the PHS and *OMHC1* in the PLS, negative values were obtained for the F_IS_ statistics, which may indicate that more heterozygotes were identified for the studied microsatellite *loci* than expected. In addition, no significant deviations within HWE were found in most *loci* (except *Ovar-DRB1* and *OMHC1* in PLS). Estimations were made, based on the polymorphism of three selected *Ovar*-MHC regions, and the genetic distance between the PLS and PHS breed was 0.444. Comparison of the genotype distribution between the studied breeds, made with the use of factorial correspondence analysis, clearly separated the two breeds (Figure 1). The best—supported number of clusters in STRUCTURE was K = 2, separating PLS from PHS Figure 2. The existing differences between the studied breeds are also presented in Figure 3.

Research showed significant differences between the breeds in the content of the analyzed milk indicators (Table 3). PLS showed a significantly higher level of SCC (305.5 × 10^3^) than PHS (142 × 10^3^). In PLS, a significantly lower percentage of lymphocytes (CD2^+^, CD8^+^, CD19^+^) and cells with the MHC II surface receptor was associated with a higher SCC level. The reverse relationship was observed in PHS. Results of the cytometric analysis of sheep milk presented in Table 4 indicate a reverse relationship between SCC and cells of the immune system (ISCs). In the samples of milk with SCC content >200 × 10^3^, a lower percentage of lymphocyte subpopulation was observed (except for MHCII^+^ cells) compared to the level <200 × 10^3^. For CD2^+^, CD4^+^, CD19^+^ lymphocytes, those differences were statistically significant. Table 5 presents the assessment of the relationship between the polymorphism of the *Ovar*-MHC regions under study and the level of SCC and ISCs in the milk of both sheep breeds. Among the *DRB1* alleles (with the highest frequency), the 488 bp allele deserves attention (Table 1). The lowest number of SCC (M = 129 × 10^3^), and a significantly higher percentage of CD2^+^, CD4^+^, CD19^+^, MHCII^+^ cells were found in the milk of sheep carrying this allele. On the other hand, the lowest (except for CD8^+^) percentage of immune system cells was observed in sheep carrying the 508 bp allele. In the case of *OMHC1*, there was no significant relationship between the identified alleles and the analyzed SCC and ISCs cells. Among the *DRB2ps* alleles, sheep carrying the 272 bp allele were characterized by the lowest percentage of immune system cells, which also had the highest level of SCC (M = 277.5 × 10^3^). It should be noted that this allele was present only in the PLS breed. A similar trend was observed in carriers of the 268 bp allele. The highest (statistically significant) percentage of the CD2^+^, CD4^+^, CD8^+^ lymphocyte subpopulation was recorded in sheep carrying the 284 bp allele (this allele was present only in the PHS breed). Molecular analysis of selected *Ovar-*MHC regions in two breeds of sheep revealed the presence of 31 *DRB1*, 24 *OMHC1*, and 24 *DRB2ps* genotypes. Table 6 shows the variability of the SCC and ISCs against the genotypes with the highest frequency. The presented data indicate a large influence of a given allele (Table 5) on the phenotypic value of the genotype. The 488 bp/488 bp homozygotes (as well as the alleles—Table 5) had a lower SCC value (M = 124 × 10^3^) and a higher percentage of ISCs compared to the other genotypes (508 bp/508 bp, 566 bp/566 bp). A similar relationship was found between the *DRB2ps* genotypes (284 bp/284 bp and 268 bp/268 bp, 268 bp/272 bp).

## 4. Discussion

### 4.1. Microsatellite Polymorphism of the MHC Genes

The usefulness of microsatellite sequences for the assessment of *Ovar*-MHC gene polymorphism in sheep has been reported in many scientific papers [69]. Paterson [31], analyzing microsatellite polymorphism in exon 2 of the *Ovar-DRB1* gene, found, in the Scottish primitive Soay sheep, the presence of 8 alleles ranging in length from 205 bp to 287 bp. Similar polymorphism was demonstrated in our own studies (Table 1 and Table 2), even though a different and longer fragment (exon 2/intron 2) of this gene was analyzed. Much greater genetic diversity of the *Ovar-DRB1* gene (14 alleles—from 148 bp to 238 bp) was reported by Worley et al. [33] in sheep of various breeds in Norway, Canada, and Alaska, Geldermann et al. [34] in German merino (16 alleles), as well as Gowane et al. [70] in Malpura (23 alleles). The *OMHC1* gene polymorphism demonstrated in this study (Table 1 and Table 2) is also confirmed in papers by other authors. Groth and Wetherall [71] identified eight *OMHC1* alleles in Australian merino; Paterson [31] identified five alleles (184–206 bp) in Soay sheep; Worley et al. [33] identified 11 alleles (178–200 bp) in sheep of various breeds; and Santucci et al. [35] identified 14 alleles (168–212 bp) in different breeds of sheep. Blattman and Beh [28], analyzing microsatellite polymorphism of the *Ovar-DRB2* pseudogene, found 11 alleles (265–295 bp) in the studied sheep, while Worley et al. [33] found more than twice as many—23 alleles (238–286 bp). Those results differ significantly from both the results of our own research and those obtained by Paterson [31], who identified only six alleles in the Soay sheep (265–283 bp). Summing up, it should be stated that high values of heterozygosity coefficients (H_E_ > 0.7) in both breeds of sheep (PHS and PLS) for all analyzed *Ovar*-MHC genes indicate that these *loci* can be used as genetic markers.

### 4.2. MHC Genes Polymorphism and Its Relations to SCC and ISCs

The analysis of the variability of the lymphocyte subpopulation in the milk of ruminants depending on the udder health has been researched by many authors. Chaffer et al. [72] showed that milk obtained from bacteria-infected cow udder quarters contained a significantly lower percentage of T lymphocytes (CD4^+^ and CD8^+^) compared to milk obtained from healthy quarters. Winter and Colditz [21], when infecting the udder of sheep with *S. epidermidis*, observed in the milk of these animals 24–72 h after injection, a significantly lower percentage of CD4^+^, CD8^+^ lymphocytes, B lymphocytes, and MHCII^+^ cells than in the milk of the control group. In other studies, Persson-Waller and Colditz [22], as early as 4 h after stimulation of the udder of sheep, *S. aureus* and *E. coli* noted a significantly lower percentage of CD8^+^ and CD4^+^ lymphocytes in the secretion of the dried udder of the experimental group compared to the control group. In own research (Table 4), sheep’s milk containing more than 200 × 10^3^/mL SCC showed a lower percentage of T (CD2^+^, CD4^+,^ CD8^+)^ and B (CD19^+^) lymphocytes than milk <200 × 10^3^/mL SCC. A different result was only obtained for MHCII^+^ cells. An increase in SCC in milk above the accepted norm of 200 × 10^3^/mL and a decrease in the percentage of lymphocytes may be associated with the developing inflammation of the mammary gland caused by pathogenic microorganisms. This thesis is confirmed by the studies of the authors cited above, who (as a result of infection of animals (*S. aureus and E. coli*) [22] or vaccination [21]) recorded a significantly lower percentage of lymphocytes compared to the control group. Different results were obtained by Taylor et al. [25] and Riollet et al. [27], who found in the milk of sheep and cows during bacterial infections of the mammary gland a greater percentage of CD4^+^, CD8^+^ lymphocytes compared to milk from the infection-free udder. Those differences in the above-cited studies and in our own research on the participation of CD4^+^ and CD8^+^ in milk may depend not only on the heterogeneous conditions of the research (breed, physiological state, lactation period, time after infection), but also on the type of microorganisms causing udder diseases. This is confirmed in the research by Soltys and Quinn [73], which shows that T lymphocytes (CD4^+^, CD8^+^) are selectively recruited in udder infection depending on the type of bacteria present in the mammary gland. B lymphocytes (CD19^+^) and cells with the MHCII receptor (mainly antigen-presenting cells) are involved in the complex process of the humoral response, i.e., the next phase after inflammation in which neutrophils play an essential role. An increase in the proportion of neutrophils in the initial phase of response to an infectious agent reduces the proportion of cells involved in the subsequent humoral response process. This conclusion is based on many scientific studies [21,22,23,24] in which animals were experimentally infected. The variability in the health status of sheep udder (SCC) depending on the identified allele of the microsatellite sequence of the *DRB1* gene (MHC class II) noted in our own research (Table 5) was reflected in the variability of the percentage composition of the analyzed ISCs in milk. Sheep, which carry the 508 bp allele (*Ovar-DRB1*), with higher SCC levels, had a significantly lower percentage of T lymphocytes (CD2^+^, CD4^+^, CD8^+^) and MHCII^+^ cells compared to these, which carry the 488 bp allele. It should be emphasized that the 488 bp allele in the PHS breed was more than 7.5 times more frequent than in the PLS breed (Table 1). Those sheep showed higher SCC levels (>200 × 10^3^/mL). Considering the above, significant differences in the percentage composition of lymphocytes in milk (Table 5), observed between carriers of the 508 bp and 488 bp alleles and related to different SCC milk levels, may indicate different susceptibility of their carriers to udder inflammation. Among the analyzed alleles of the *Ovar-DRB2*, the presence of the 272 bp allele in the sheep genotype was more frequently related to the higher SCC levels (>200 × 10^3^/mL), in contrast to the 284 bp allele, which was found in sheep that showed lower SCC levels (<200 × 10^3^/mL). It should also be emphasized that the 272 bp allele was present only in PLS, which are more susceptible to mastitis [47,48], while the 284 bp allele was identified only in PHS, more resistant to this disease [47,48]. Such suggestions regarding the link of certain alleles with SCC are confirmed by the identified genotypes presented in Table 6.

## 5. Conclusions

Studies showed statistically significant differences in the health condition of the mammary gland (demonstrated by SCC levels in milk) of the PHS and of the PLS. Varied numbers of SCC in the milk of these sheep breeds were reflected in the variability of the percentage of cells in the immune system. It was found that in PHS, the percentage of lymphocyte subpopulation in milk was significantly higher than in PLS. A higher percentage of lymphocytes in milk was associated with a lower level of SCC. Molecular analyses identify significant microsatellite polymorphism of the three studied *Ovar*-MHC regions in the two sheep breeds. This polymorphism and the estimated high values of expected heterozygosity coefficients for the majority of the analyzed fragments of *Ovar-*MHC genes indicate the possibility of searching for genetic markers of sheep resistance to diseases among alleles of these genes. Based on the analysis of the values of Ho, H_E_, and PIC parameters, it can be concluded that PHS sheep were characterized by greater heterozygosity than PLS sheep. Significant differences in SCC and percentage of ISCs depending on the identified alleles of the *Ovar-*MHC genes were also shown. The 488 bp (*DRB1*) and 284 bp (*DRB2*) alleles were more frequently found in sheep with low SCC levels (<200 × 10^3^/mL), while the carriers of the 508 bp (*DRB1*) and 272 bp (*DRB2*) alleles showed higher SCC levels (>200 × 10^3^/mL), which may suggest higher susceptibility to mastitis. For the *OMHC1* alleles, no clear relationship was observed between them and SCC and activity of ISCs. Previous studies have shown that resistance to different diseases is influenced by many factors, but one of the most important is genetic variation. Despite many scientific achievements, there are still difficulties in precisely determining the role that genetic variation plays in the individual shaping of the immune response process induced by pathogens. The results of many studies, however, indicate that further improvement and standardization of the research methods should enable both a better understanding of the complex defense processes, and the determination of such genetic markers that can be successfully used in breeding practice.

## Figures and Tables

**Table 1:** Frequency of the *Ovar-*MHC alleles in the studied sheep breed.

*Locus*/Breed	Allele Frequency (%)								
*Ovar-DRB1* (bp)	488	508	516	520	526	530	540	566	590
PHS	46.1	11.0	10.2	1.6	7.5	1.6	5.1	13.4	3.5
PLS	6.1	32.0	15.0	3.7	4.4	1.4	8.8	28.6	-
*OMHC1* (bp)	188	190	192	194	196	202	208		
PHS	8.3	8.7	32.3	29.0	4.0	10.0	7.7		
PLS	4.9	12.7	6.8	35.7	25.6	14.3	-		
*Ovar-DRB2* (bp)	262	268	272	274	276	284	290		
PHS	17.2	32.8	-	20.9	-	22.0	7.1		
PLS	7.1	38.8	25.9	12.9	12.9	-	2.4		

PLS—Polish Lowland Sheep; PHS—Polish Heath Sheep. *Ovar-*MHC—Major Histocompatibility Complex in sheep.

**Table 2:** Analysis of genetic variability of the *Ovar*-MHC microsatellite *loci* in the Polish Heath Sheep (PHS) and the Polish Lowland Sheep Żelazna variety (PLS).

Breed.	PHS							PLS						
*Locus*	n	No. of Alleles	H_O_	H_E_	PIC	HW ^b^	F_is_	n	No. of Alleles	H_O_	H_E_	PIC	HW ^b^	F_is_
*Ovar-DRB1*	126	9	0.75	0.74	0.71	NS	−0.01060	147	8	0.58	0.78	0.75	***	0.25184
*OMHC1*	149	7	0.80	0.78	0.75	NS	−0.02306	154	6	0.84	0.77	0.73	**	−0.10286
*Ovar-DRB2*	133	5	0.74	0.77	0.73	NS	0.03869	147	6	0.70	0.75	0.71	NS	0.70440
Mean		7	0.76	0.76	0.73		0.00169 ^a^		6.67	0.71	0.77	0.73		0.07445 ^a^
±SD		1.63	0.03	0.02	0.02				0.94	0.11	0.01	0.02		

n—number of analyzed individuals, H_O_—observed heterozygosity, H_E_—expected heterozygosity, PIC—Polymorphism Information Content, F_IS_—inbreeding coefficient, ^a^ multilocus F_IS_, ^b^ Hardy-Weinberg equilibrium significance level: *** significant at *p* ≤ 0.001, ** significant at *p* ≤ 0.01, NS—insignificant.

**Table 3:** Median of somatic cells number and proportions of immune cells in the milk of the Polish Heath Sheep (PHS) and the Polish Lowland Sheep Żelazna variety (PLS).

Breed	Median	Median of Immune Cells (%)				
	SCC × 10^3^	CD2^+^	CD4^+^	CD8^+^	CD19^+^	MHCII^+^
PHS	142.0 ^A^	55.0 ^A^	22.8	31.0 ^A^	18.0 ^a^	58.5 ^A^
PLS	305.5 ^B^	43.5 ^B^	21.0	17.3 ^B^	14.0 ^b^	40.0 ^B^

Significant values—different letters (^ab^—*p* ≤ 0.05, ^AB^—*p* ≤ 0.01).

**Table 4:** The relation between the number of somatic cells and the proportion of the lymphocyte subpopulation and MHCII cells in the milk of studied sheep.

SCC × 10^3^	n	Median of Immune Cells (%)				
		CD2^+^	CD4^+^	CD8^+^	CD19^+^	MHCII^+^
<200	296	53.5 ^A^	24.0 ^A^	25.0	18.0 ^A^	45.0
>200	312	47.0 ^B^	19.3 ^B^	22.0	14.0 ^B^	53.0

Significant values—different letters (^AB^—*p* ≤ 0.01).

**Table 5:** Median of somatic cells number and proportions of immune cells in the milk of the Polish Heath Sheep (PHS) and the Polish Lowland Sheep Żelazna variety (PLS) in relation to the analyzed *Ovar-*MHC regions.

*Locus*/Allele (bp)		n	Median	Median of the Immune Cells (%)				
			SCC × 10^3^	CD2^+^	CD4^+^	CD8^+^	CD19^+^	MHCII^+^
*Ovar-DRB1*	488	133	129.0	54.5 ^aA^	25.5 ^aA^	30.0 ^aA^	17.6	50.0 ^a^
	508	122	232.5	44.5 ^B^	17.0 ^B^	18.8 ^B^	11.0	37.0 ^b^
	516	70	256.5	53.0 ^a^	21.3 ^a^	21.8 ^a^	19.0	38.3
	566	118	254.0	46.5 ^B^	20.0 ^a^	16.3 ^B^	18.0	46.0
*OMHC1*	192	118	171.0	52.0	21.5	26.0	15.0	47.0
	194	195	211.0	53.5	22.8	25.0	17.0	52.0
	196	88	329.0	46.8	22.8	19.0	15.0	41.3
	202	74	193.0	49.3	19.8	21.8	14.0	48.0
*Ovar-DRB2ps*	268	202	206.5	47.0 ^a^	21.5 ^a^	21.0 ^ab^	16.5	47.0 ^a^
	272	72	277.5	37.0 ^bA^	15.0 ^A^	15.3 ^b^	13.5	30.0 ^bB^
	274	92	265.0	50.0 ^a^	19.0 ^a^	25.8 ^a^	18.0	59.5 ^aA^
	284	59	156.0	56.5 ^aB^	27.0 ^aB^	31.0 ^a^	16.5	45.0 ^a^

Significant values—different letters (^ab^—*p* ≤ 0.05, ^AB^—*p* ≤ 0.01).

**Table 6:** Median of somatic cells number and proportions of immune cells in the milk of the Polish Heath Sheep (PHS) and the Polish Lowland Sheep Żelazna variety (PLS) in relation to the selected *Ovar-*MHC genotypes.

Genotype (bp/bp)		n	Median	Median of Immune Cells (%)				
			SCC × 10^3^	CD2^+^	CD4^+^	CD8^+^	CD19^+^	MHCII^+^
*Ovar-DRB1*	488/488	19	124.0	55.5	31.0	26.0	21.0	60.0
	508/508	24	219.0	37.5	17.0	17.5	10.0	32.5
	566/566	22	231.5	42.0	21.5	8.0	19.0	59.0
*OMHC1*	192/194	31	159.0	46.3	20.0	16.0	21.5	47.0
	194/196	36	282.0	54.8	29.5	20.0	15.3	44.3
	194/202	23	226.0	50.0	20.0	25.0	14.0	49.0
*Ovar-DRB2ps*	268/268	34	221.0	43.0	28.5	22.0	18.0	63.5 ^A^
	268/272	17	1176.0	37.5 ^a^	15.0	8.0	13.0	23.5 ^B^
	284/284	10	132.0	68.0 ^b^	35.2	26.2	17.0	42.5

Significant values—different letters (^ab^—*p* ≤ 0.05, ^AB^—*p* ≤ 0.01).
